# Structural basis for the inhibition mechanism of LAT1-4F2hc complex by JPH203

**DOI:** 10.1038/s41421-024-00697-6

**Published:** 2024-07-02

**Authors:** Ziwei Hu, Renhong Yan

**Affiliations:** Department of Biochemistry, School of Medicine, Key University Laboratory of Metabolism and Health of Guangdong, Institute for Biological Electron Microscopy, Southern University of Science and Technology, Shenzhen, Guangdong China

**Keywords:** Cryoelectron microscopy, Tumour biomarkers

Dear Editor,

The L-type amino acid transporter 1 (LAT1 or SLC7A5), coupled with 4F2hc (or SLC3A2), facilitates the transport of large neutral amino acids and thyroid hormones across cell membranes in a sodium-independent exchange manner^1–3^. LAT1 emerges as a significant anti-cancer target due to its role in suppressing the proliferation of various cancer cells when pharmacologically inhibited or knocked down/knocked out^4–7^. Its counterpart, LAT2, shares 54% sequence identity and 71% sequence similarity with LAT1 (Supplementary Fig. S1) and exhibits a broader substrate range, encompassing small amino acids, primarily found in normal tissues^6,8,9^. JPH203 (or KYT-0352), a triiodothyronine (T3)-derived amino acid, stands out for its ability to inhibit tumor growth in multiple cancer cell lines by selectively inhibiting LAT1’s transport activity rather than LAT2^10^. Currently, JPH203 is being evaluated in a Phase II clinical trial (UMIN000034080) for the treatment of biliary tract cancer. This follows a phase I clinical trial (UMIN000016546) that demonstrated the safety and efficacy of JPH203^11^. Despite advancements in understanding LAT1 and LAT2 structures^8,12–15^, the precise molecular mechanism underlying JPH203’s specific inhibition of the LAT1**–**4F2hc complex remains incompletely elucidated. In this study, we determined the cryo-EM structure of the LAT1**–**4F2hc complex bound with the JPH203 inhibitor, shedding light on the intricate details of its specific inhibition mechanism.

In our previous study, we attempted to incubate JPH203 with concentrated protein to determine the structure of the LAT1**–**4F2hc complex bound with JPH203, but were unsuccessful^12^. This time, we optimized the incubation conditions using 500 μM JPH203 with the eluent from the size-exclusion chromatography, followed by concentration for cryo-EM sample preparation (Supplementary Fig. S2b). After several rounds of 3D refinement, we successfully revealed the outward-facing structure of the LAT1**–**4F2hc complex bound with JPH203 at a resolution of 3.30 Å (Fig. 1a; Supplementary Figs. S2, S3 and Table S1). Additionally, we applied focused refinement to enhance the density of the corresponding JPH203 (3.25 Å) and support the final model building (Supplementary Figs. S2d, S3a). The details of cryo-EM sample preparation, data collection and processing, and the atomic model building can be found in the Supplementary Materials and Table S1.

**Fig. 1:**
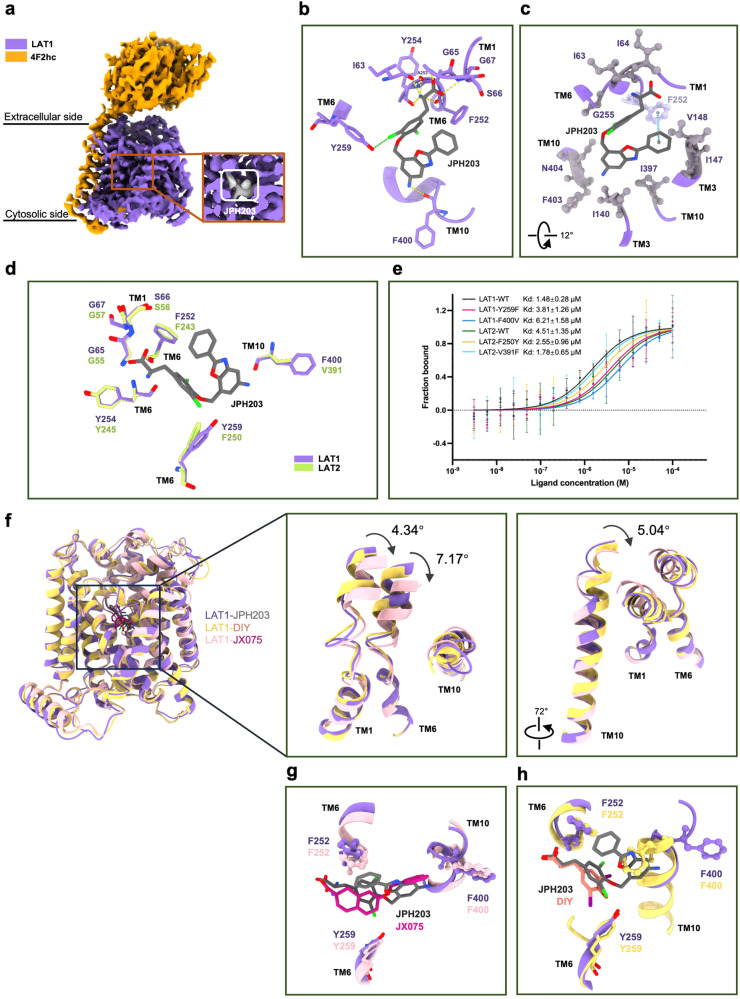
Overall structure of the LAT1–4F2hc bound with JPH203. **a** Cryo-EM map of the full length of LAT1**–**4F2hc complex with JPH203. 4F2hc and LAT1 with JPH203 (dim gray) are represented in orange and medium purple,respectively. The inserts in cryo-EM map of the human LAT1**–**4F2hc complex. The density corresponding to JPH203 is color-coded in dim gray. **b** The hydrogen bond network at the JPH203-binding pocket of the LAT1 structure. Hydrogen bonds clusters are indicated by yellow dashed lines. Halogen bonds is represented by green dashed line. The gating residue Phe252 is colored taro purple, and steel blue dashed line represents T-shaped π–π interaction. **c** Hydrophobic interaction formed among Ile63, Ile64, Ile140, Ile147, Val148, Gly255, Ile397, Phe403, and Asn404. **d** Comparison between LAT1 bound with JPH203 and homologous structure LAT2. **e** The binding affinity of JPH203 was evaluated in relation to the WT LAT1**–**4F2hc complex and LAT2**–**4F2hc complex, as well as its mutations, Y259F and F400V in LAT1, F250Y and V391F in LAT2. **f**, **g** Comparison of the binding mode of the three inhibitors. JX075, JPH203, and Diiodo-tyr are colored yellow, dim gray and salmon, respectively. **h** Structural comparison among the outward-facing structure of LAT1 bound with JPH203 and the outward-facing occluded structure of LAT1 bound with JX075 or Diiodo-Tyr. The movement of TM1, TM6 and TM10 is shown in the middle and right panel, respectively. JPH203, JX075, and Diiodo-Tyr are colored purple, pink, and yellow, respectively.

Structurally, JPH203 comprises two distinct sections: a hydrophilic head group with a phenylalanine backbone and a hydrophobic tail group with a 5-amino-2-(3-aminophenyl) benzoxazole backbone. In our structural analysis, we observed that JPH203 binds within the traditional substrate-binding pocket, akin to previously determined inhibitors such as Diiodo-Tyr or JX-075, JX-078, and JX-119 in LAT1 (Fig. 1b, c)^13^. The α-amino group and α-carboxyl group of the head play pivotal roles in stabilizing the molecule, forming a hydrogen bond network with the main chain atoms in the unwound region of TM1 and TM6 in LAT1 (Fig. 1b). Furthermore, the chloride atom on the bi-halogenated tyrosine core of JPH203 forms a halogen bond with Tyr259. The hydrophobic tail moiety snugly fits into a hydrophobic pocket, which is formed by specific amino acids: Ile63, Ile64, and Ile68 on TM1; Ile140, Ile147, Ile148 on TM3; Phe252 and Gly255 on TM6; along with Ile397, Phe400, and Phe403 on TM10 (Fig. 1c). Specifically, the amino group of the tail forms a hydrogen bond with the main chain of Phe400 on TM10. Moreover, the benzene ring of the tail group engages in a T-shaped π–π interaction with the side chain of Phe252 (Fig. 1c). These intricate interactions collectively contribute to enhancing the structural stability and specificity of JPH203 as an inhibitor.

To further illuminate the selective mechanism of JPH203 towards LAT1 over LAT2, we utilized the structure of LAT1 bound with JPH203 to generate a LAT2 model by SWISS-MODEL and conducted a structural comparison (Fig. 1d; Supplementary Fig. S4). An essential observation arose concerning the residues interacting with JPH203. Specifically, Phe400 in LAT1, crucial for stabilizing the interaction with JPH203, corresponds to Val391 in LAT2. Similarly, Tyr259 in LAT1, another notable residue in the JPH203 interaction, corresponds to Phe250 in LAT2, resulting in the loss of the halogen interaction with JPH203 (Fig. 1d).

To validate these observations, we conducted specific mutations at positions Phe400 and Tyr259 in LAT1 and assessed their impact on the binding affinity of JPH203 using microscale thermophoresis (MST) assays (Fig. 1e). The results indicated that the mutations F400V (6.21 ± 1.58 μM) and Y259F (3.81 ± 1.26 μM) notably decreased the binding affinity of JPH203 to wild-type (WT) LAT1 (1.48 ± 0.28 μM) (Fig. 1e; Supplementary Fig. S5). This weakened binding may contribute to the observed reduced inhibitory effectiveness of JPH203 against LAT2. Surprisingly, the binding affinities of V391F (1.78 ± 0.65 μM) and F250Y (2.55 ± 0.96 μM) in LAT2 to JPH203 shows substantial improvement compared to the WT LAT2 (4.51 ± 1.35 μM) (Fig. 1e). These findings underscore the significance of Phe400 and Tyr259 in LAT1 for the high selectivity of JPH203, offering valuable insights into the molecular basis of its specificity.

Moreover, we conducted a comparative structural analysis of the JX075, Diiodo-Tyr and JPH203 in LAT1 (Fig. 1f–h). Interestingly, in the JPH203-bound structure, the side chain of Phe400 on TM10 is displaced, resembling the pattern observed with JX075 inhibitors, in contrast to the conformation seen in the Diiodo-Tyr bound structure. However, the gating residue of Phe252 in the JPH203-bound structure is closer to that in Diiodo-Tyr bound structure. Our previous study suggested that the extended tail of JX075 inhibitor might hinder the transition from the outward-occluded to the outward-open conformation and could also partially disrupt the secondary structure of TM3 and TM10. However, in the JPH203-bound structure, the secondary structure of TM10 is disrupted, while the TM3 remains fully folded, suggesting a different inhibition compared with the JX075. It appears that the elongated tails of JPH203 prevent the movement of Phe400, leading to distinct conformational changes compared to those induced by Diiodo-Tyr or 2-amino-2-norbornanecarboxylic acid (BCH). Examining the movements of TM1, TM6, and TM10 across JPH203, Diiodo-tyr, and JX075, distinct conformational differences become apparent among these three structures. Unlike the JX075-bound configuration, the JPH203-bound structure exhibits a 4.34° shift in TM1, along with a rotation in the TM6 domain (Fig. 1f). Further analysis revealed that TM10 in the JPH203-bound structure demonstrates a notable rotation compared to both the JX075 and Diiodo-tyr bound structures, possibly indicating the detailed working mechanism of LAT1.

In summary, the high-resolution structure of the LAT1**–**4F2hc bound to JPH203, as elucidated in our study, provides a structural basis for the rational design of selective potent inhibitors to LAT1. This advancement holds the potential to pave the way for the development of more effective therapeutic strategies.

### Supplementary information

Supplementary Figure S1-S5 and Table S1

## Data Availability

The structures of the LAT1**–**4F2hc complex bound with JPH203 (PDB: 8XPU, whole map: EMD-38561, focused map: EMD-39897) have been deposited to the Protein Data Bank (https://www.rcsb.org) and the Electron Microscopy Data Bank (https://www.ebi.ac.uk/pdbe/emdb/), respectively. Correspondence and requests for materials should be addressed to R.Y. (yanrh@sustech.edu.cn).
