# Supplementary material for: Structural basis for the inhibition mechanism of LAT1-4F2hc complex by JPH203
Source: Cell Discov. 2024 Jul 2;10:73. doi: 10.1038/s41421-024-00697-6 (PMC11220031; doi:10.1038/s41421-024-00697-6)
Supplement: Supplementary file 1 — Supplementary Figure S1-S5 and Table S1 [file 41421_2024_697_MOESM1_ESM.pdf]

Supplementary Materials for

Structural basis for the inhibition mechanism of LAT1-4F2hc complex by JPH203

Ziwei Hu[1], Renhong Yan[1*]

[1] Department of Biochemistry, School of Medicine, Key University Laboratory of Metabolism and Health of Guangdong, Institute for Biological Electron Microscopy, Southern University of Science and Technology, Shenzhen, 518055, Guangdong, China.

Correspondence to: yanrh@sustech.edu.cn

This PDF file includes:

Materials and Methods
Supplementary Figures. S1 to S5
Tables S1

Materials and Methods

Protein expression and purification

The cDNAs corresponding to the full-length human LAT1 (accession number: NM_003486.7) and 4F2hc (isoform b, accession number: NM_001012662.2) were individually subcloned into pCAG vectors. LAT1 was modified to include an N-terminal FLAG tag, while 4F2hc was fused with a 10× His tag at the C-terminus, utilizing standard PCR techniques. These recombinant proteins were overexpressed in HEK 293F mammalian cells. The cells were incubated at 37 ℃ in a Multitron-Pro shaker (Infors) at 130 rpm, in an atmosphere containing 5% CO_2. Once the cell density reached approximately 2.0×10^6 cells/mL, the cells were transiently transfected with the plasmids. These plasmids were prepared using the GoldHi EndoFree Plasmid Maxi Kit (CWBIO). For co-expression of the LAT1-4F2hc complex, a mixture of 0.75 mg of each LAT1 and 4F2hc plasmid was combined with 3 mg of polyethylenimines (PEIs, Polysciences) in 50 mL of fresh medium, and incubated for 15 minutes prior to addition to the cell culture. The cells were then cultivated for 60 hours.

For purification of the LAT1-4F2hc complex, the harvested cells were collected with centrifuge jars at 3,500×g, and then suspended in a buffer containing 25 mM HEPES (pH 7.5), 50 mM NaCl, and a trio of protease inhibitors: aprotinin (1.3 μg/mL, AMRESCO), pepstatin (0.7 μg/mL, AMRESCO), and leupeptin (5 μg/mL, AMRESCO). The cells were then treated with 1% (w/v) LMNG (Anatrace) and 0.1% (w/v) cholesteryl hemisuccinate Tris salt (CHS, Anatrace) at 4 ℃ for 2 hours. Subsequently, the mixture was centrifuged at 13,000× g for 50 minutes to remove cell debris. The clear supernatant was applied to an anti-FLAG M2 affinity resin (Sigma), and the resin was washed with 40 mL buffer containing 25 mM HEPES (pH 7.5), 150 mM NaCl, and 0.01% GDN (w/v). The proteins were eluted using 20 mL the same wash buffer supplemented with 0.2 mg/mL FLAG peptide. Further purification was achieved using a Ni-NTA affinity resin (Qiagen), with 20 mL wash and 10 mL elution buffers composed of the aforementioned wash buffer plus 10 mM and 300 mM imidazole, respectively. Finally, the protein complex was subjected to size-exclusion chromatography (Superose 6 Increase 10/300 GL, GE Healthcare) in a buffer containing 25 mM HEPES (pH 7.5), 150 mM NaCl, and 0.01% GDN. The peak fractions were collected and concentrated for subsequent EM analysis.

Cryo-EM sample preparation and data acquisition

The LAT1-4F2hc complex was incubated with final concentration 500 μM of the inhibitor JPH203 (Selleck) for 1 hour at 4℃. Subsequently, it was purified and concentrated to approximately 9 mg/mL using 50 kDa cut-off concentrator at 4,500×g, prior to being applied to the grid. The glow discharge grid settings were as follows: current 15 mA, hold for 30 seconds, wait for 30 seconds. For electron microscopy, small aliquots (3.3 μL) of this protein complex were carefully applied onto glow-discharged holey grids with amorphous alloy film (CryoMatrix, Au R1.2/1.3, 300 mesh). Following a 3.5-second blotting process, these grids were rapidly plunged into liquid ethane, ensuring cryogenic preservation with liquid nitrogen, using a Vitrobot (Mark IV, Thermo Fisher Scientific). The prepared cryo-EM samples were then loaded into a Titan Krios electron microscope, which operates at 300 kV. This state-of-the-art microscope was outfitted with a Gatan K3 detector and a GIF Quantum energy filter. Image acquisition was automated via AutoEMation, with the energy filter slit width maintained at 20 eV. The samples were imaged under a range of defocus levels, from -1.4 μm to -1.8 μm, in super-resolution mode at a nominal magnification of 81,000×. Each movie stack was recorded over a total exposure time of 2.998 seconds, divided into 32 frames with 0.094 seconds exposure per frame. The total electron dose was approximately 50 electrons per Ångström squared (e-/Å2) for each stack. Post-acquisition, the movie stacks were processed for motion correction using MotionCor2. The images were binned by a factor of 2, resulting in an effective pixel size of 1.095 Å/pixel. Dose-weighting adjustments were made to the images. Additionally, Gctf was employed to accurately estimate defocus values.

Homology Model

The protein sequence of LAT2 from Homo sapiens was retrieved from the UniProt database (Accession Number: Q9UHI5) and consists of 535 amino acid residues. The outward-occluded cryo-EM structure of LAT1-4F2hc (PDB: 7DSQ) from Homo sapiens, used as a template, was obtained from the Protein Data Bank (https://www.rcsb.org/structure/7DSQ). The model was generated using the SWISS-MODEL server (https://swissmodel.expasy.org), and the best model was selected based on QMEAN and Z-score evaluations from ProSA-web. The final comparison between LAT1 and LAT2 was visualized using ChimeraX v1.6.1.

Microscale Thermophoresis (MST)

Wild-type (WT) and mutant full-length coding sequences of LAT1-4F2hc (Y259F and F400V) and LAT2-4F2hc (F250Y and V391F) were cloned into a GFP-pCAG vector. These plasmids were then transformed into HEK-293F cells for protein expression. The subsequent extraction and purification of the protein followed the same procedures as above described.

MST experiments were conducted using a Monolith NT.115Pico optics system (NanoTemper Technologies) [1], [2]. The settings for these experiments were 100% LED power and 20% IR-laser power, with laser 'on' and 'off' times set at 30 seconds and 5 seconds, respectively. The GFP-LAT1-4F2hc protein was used at a final concentration of 100 nM. JPH203 (Selleck), was initially dissolved in DMSO to create a 2 mM stock solution. A two-fold serial dilution series of the unlabeled JPH203 in DMSO was prepared. For each assay point, 1 μL of a specific dilution was mixed with 19 μL of the GFP-recombinant LAT1-4F2hc protein solution. This approach allowed the final JPH203 concentration to range from 100 μM to 3.05 nM, while consistently maintaining the DMSO concentration at 5% across all samples. The prepared samples were loaded into hydrophilic capillaries (NanoTemper Technologies) for MST measurement. The binding affinity assay was independently replicated at least three times. Data obtained from these experiments were combined and analyzed using the MO. Affinity Analysis v2.3 software. This analysis provided several key parameters, including the dissociation constant (Kd) value, Kd confidence interval, response amplitude, and the signal-to-noise ratio.

Data processing

Particle selection from the micrographs was performed automatically using blob picking and template picking techniques in CryoSPARC v4.4.1[3,4]. The process began with a round of 2D classification, followed by the selection of a subset of high-quality particles for advanced processing. Subsequent steps included 3D reconstruction via Ab-initio Reconstruction, which yielded an initial coarse resolution model of the complex. To achieve better resolution, the Heterogeneous Refinement and Non-uniform refinement method was employed. Throughout this process, the current resolution was iteratively assessed via Fourier Shell Correlation, alongside other diagnostic data. To further enhance the map quality, especially in the ligand (JPH203) region, a combination of Local CTF Refinement and Local Refinement techniques was applied. These techniques focused on applying specific masks to target and improve resolution in the ligand

region. The final resolution of map was estimated using the gold-standard Fourier shell correlation criterion of 0.143 [5,6]. Additional details regarding data collection and processing can be found in Supplementary Figure S2 and Table S1.

Model building and structure refinement

The atomic models of the LAT1-4F2hc complex in complex with JPH203 were constructed based on the corresponding Cryo-EM maps. For this construction, we utilized the previously determined structure of the LAT1-4F2hc complex bound with Diiodo-tyrosine (PDB ID: 7DSQ) as a template. The initial models were subject to further manual refinement using the Coot software (v0.9.8.1) [7], with particular focus on accurately representing the chemical properties of each amino acid residue. To achieve precise refinement, we employed structural real-space refinement techniques using the software Phenix (v1.20.1-4487)[8]. During this refinement phase, we incorporated secondary structure and geometric constraints to avoid potential overfitting. To ensure the integrity of the model and mitigate the risk of overfitting, the refinement process was conducted in a stepwise manner. Initially, the model was refined against one of the two independent half maps, adhering to the principles of gold-standard 3D refinement. Following this, the refined model was rigorously validated against the other half map to ensure its accuracy and reliability. Comprehensive statistical data related to the procedures of data collection, 3D reconstruction, and model refinement are detailed in Table S1.

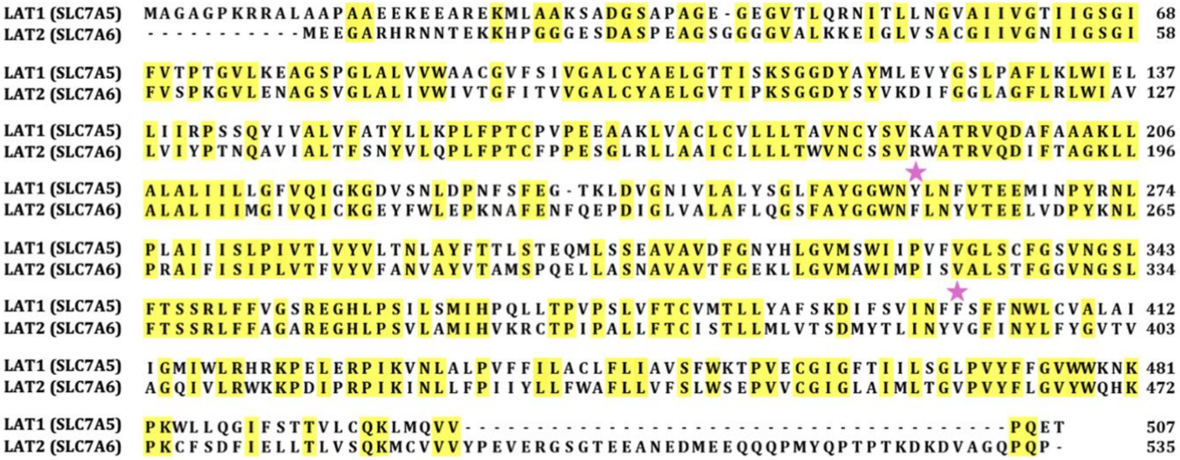

Supplementary Fig. S1. Sequence alignment of human LAT1 and LAT2. The sequence alignment of LAT1 and LAT2 homologues from *Homo sapiens*, conducted using the MAFFT algorithm. Residues that are identical or conserved across two sequences are highlighted in yellow. The sequences correspond to the following UNIPROT accession numbers: LAT1 (Q01650) and LAT2 (Q9UHI5).

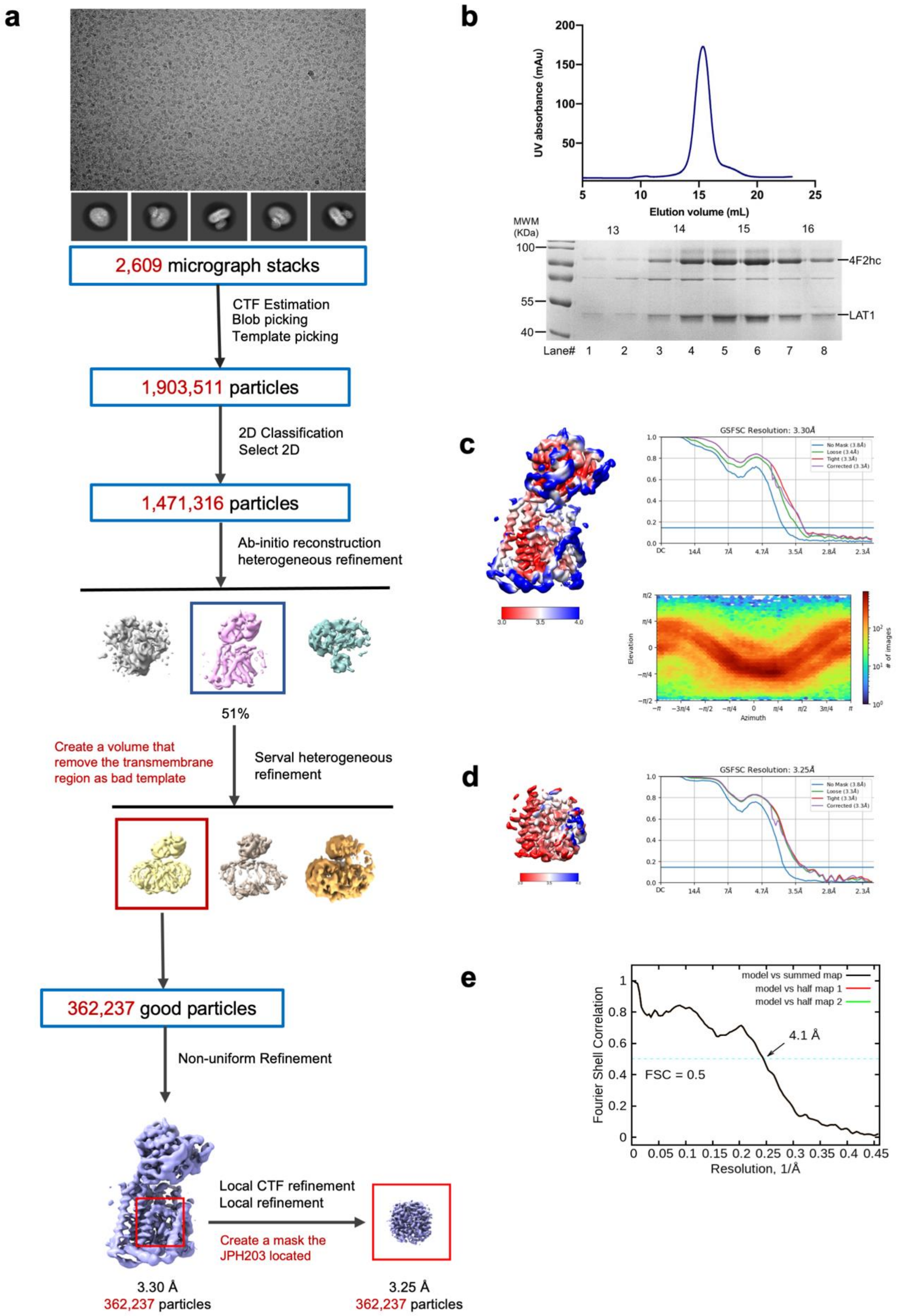

Supplementary Fig. S2. Cryo-EM reconstruction of LAT1-4F2hc bound with JPH203. (**a**) Flowchart for cryo-EM data processing. (**b**) Representative size exclusion chromatography (SEC)

purification of the LAT1-4F2hc complex. The protein complex was purified in the presence of 0.01% GDN. Inset: on the top, the SEC purification diagram of the full-length human LAT1-4F2hc complex; on the bottom, SDS-PAGE visualized by Coomassie blue staining. MWM, molecular mass marker. (**c**) Cryo-EM density maps for LAT1-4F2hc bound with JPH203. On the left, local resolution map of the overall structure; On the top right, fourier shell correlation (FSC) curve calculated using two independent half maps. Resolution was estimated using the FSC=0.143 cutoff. On the low right, Euler distribution of the refined particles. (**d**) Local refinement cryo-EM density maps for LAT1-4F2hc bound with JPH203. On the left, Local resolution map for the local structure; On the right, FSC curve calculated using two independent half maps. (**e**) FSC curve of the refined model versus the overall structure that it is refined against (black); of the model refined against the first half map versus the same map (red); and of the model refined against the first half map versus the second half map (green). The small difference between the red and green curves indicates that the refinement of the atomic coordinates did not suffer from overfitting.

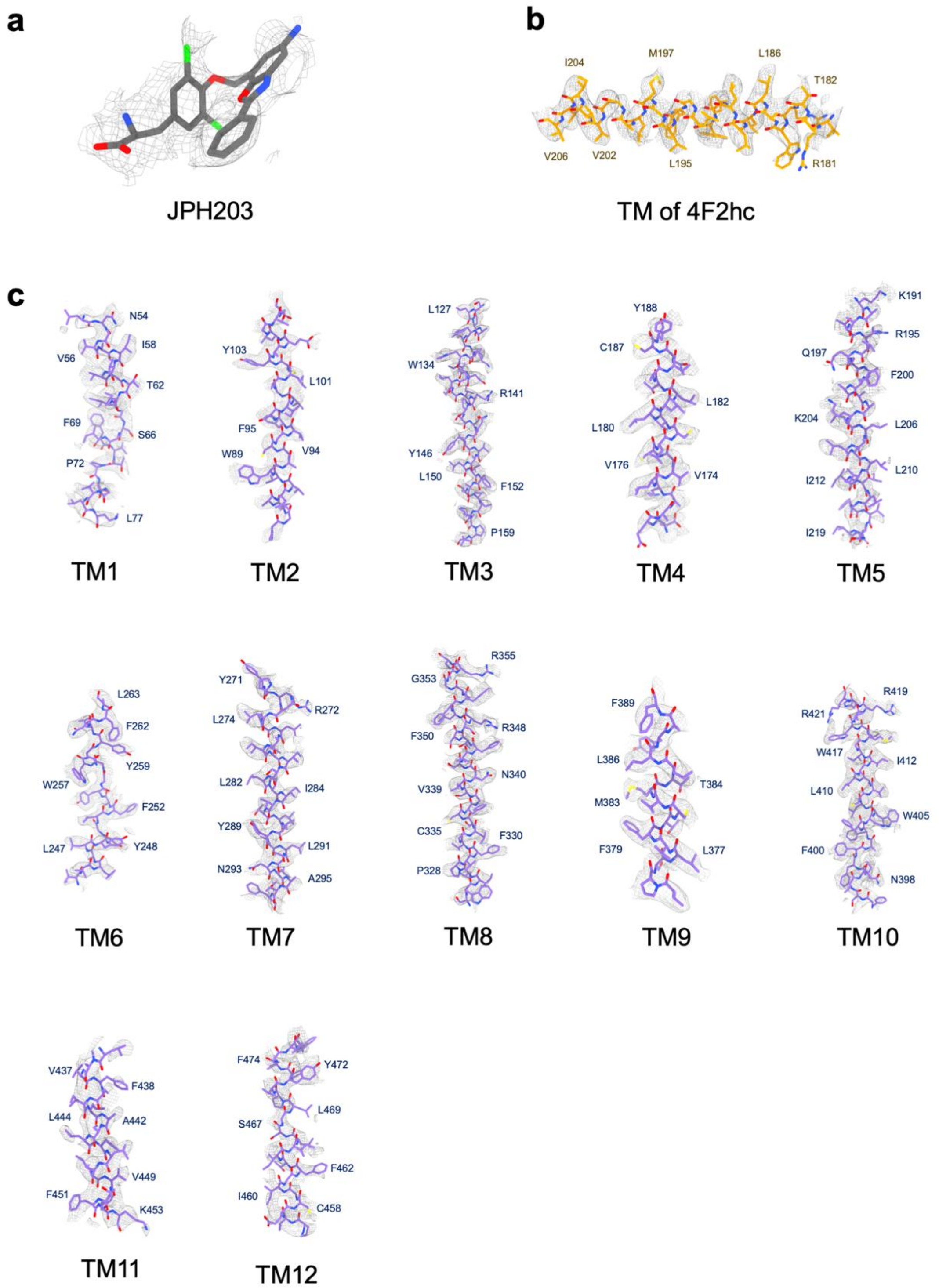

Supplementary Fig. S3. Cryo-EM density maps of the LAT1-4F2hc bound with JPH203. (**a**) Cryo-EM density maps for the LAT1-4F2hc bound with JPH203 shown at threshold of 7σ. (**b-c**) Cryo-EM density maps for the transmembrane helix of LAT1-4F2hc shown at threshold of 4σ.

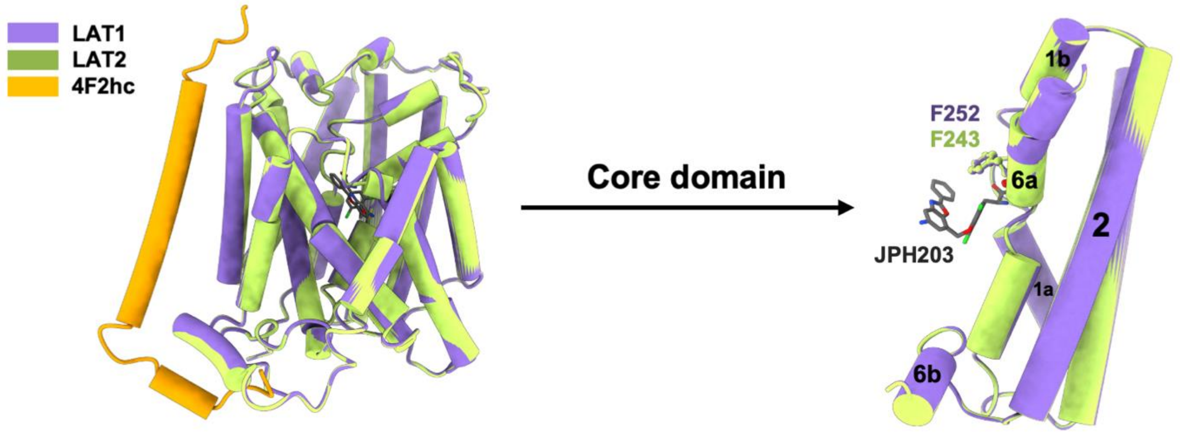

Supplementary Fig. S4. The asymmetrical movement of LAT1 bound with JPH203 and LAT2. A comparison between the outward-occluded structure of LAT1 complexed with JPH203 and the homology-modeled structure of LAT2. LAT1 is depicted in medium purple, whereas LAT2 is represented in green.

a

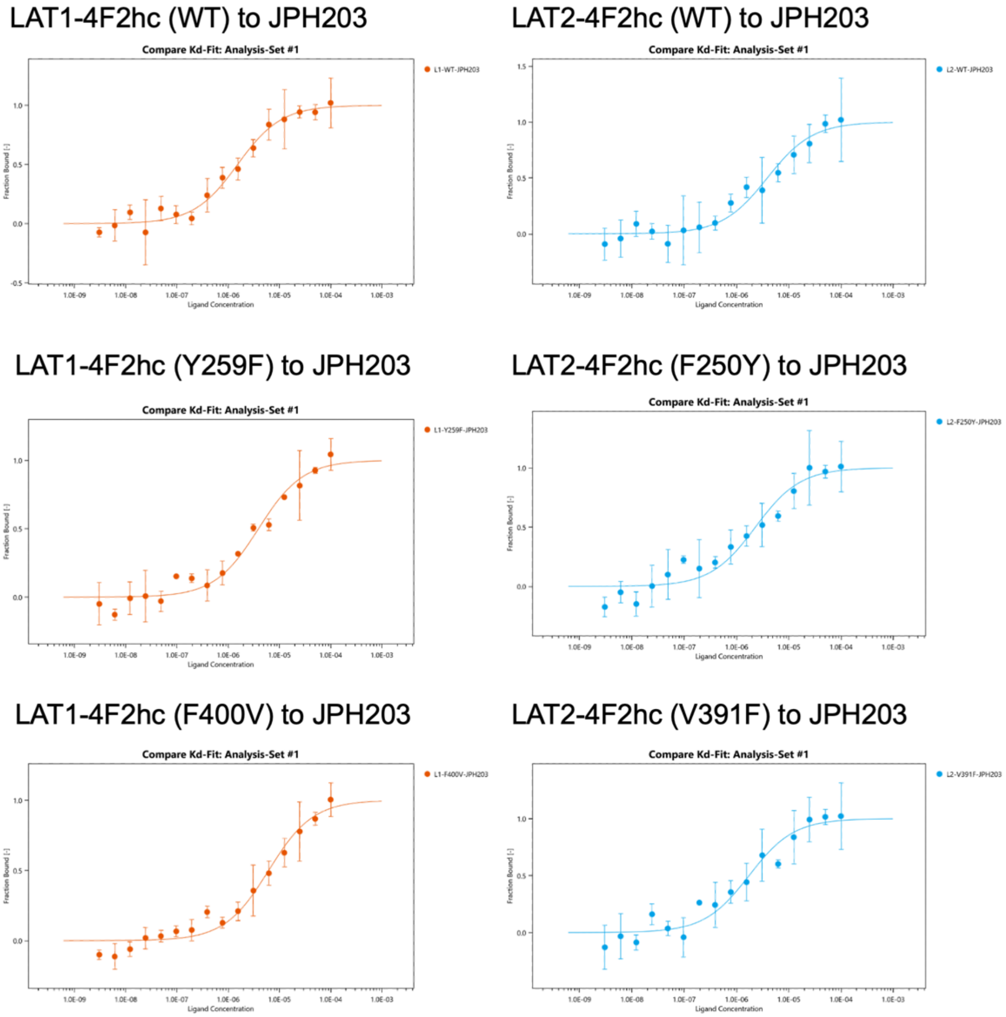

b

Group	Kd (M)	Kd Confidence	Unbound	Bound	n	Signal to Noise
LAT1-WT to JPH203	1.5E-06	2.77419E-07	888.09621	879.60019	3	17.94813163
LAT1-Y259F to JPH203	3.8E-06	1.25638E-06	889.69821	879.70423	3	10.80385818
LAT1-F400V to JPH203	6.2E-06	1.57905E-06	889.42823	876.11963	3	14.90589942
LAT2-WT to JPH203	4.5E-06	1.3502E-06	886.4057	882.0511	3	12.106875
LAT2-V391F to JPH203	1.8E-06	6.45978E-07	888.14679	883.16288	3	9.326910262
LAT2-F250Y to JPH203	2.5E-06	9.57807E-07	888.62528	882.44206	3	9.155227065

Supplementary Fig. S5. Binding affinity of JPH203 with LAT1-4F2hc and LAT2-4F2hc complexes, as well as their mutations (a) Fitting analysis for each experimental group. (b) Kd values, Kd confidence intervals, and signal-to-noise ratios for each MST group. Each experiment was conducted in triplicate.

Supplementary Table S1.

Cryo-EM data collection, refinement and validation statistics

**Data collection**	
EM equipment	Titan Krios (Thermo Fisher Scientific)
Voltage (kV)	300
Detector	Gatan K3 Summit
Energy filter	Gatan GIF Quantum, 20 eV slit
Magnification	81,000×
Pixel size (Å)	1.095
Symmetry imposed	C1
Electron dose (e-/Å2)	50
Defocus range (µm)	-1.4 to -1.8
Number of collected micrographs	2,609
Map Resolution (Å)	3.30
FSC threshold for resolution determination	0.143
Map resolution range (Å)	2.5-5
3D Reconstruction	
Software	CryoSPARC (v4.4.1)
Initial model used for refinement	1,471,316
Final particles used	363,237
Model resolution (Å)	3.73
FSC threshold	0.5
Model resolution range (Å)	3.21
B factor ($Å^2$)	-167.7
Refinement	
Software	Phenix (v1.20.1-4487)
Model composition	LIG:1 NAG:8
Protein residues	934
Non-hydrogen atoms	7,391
CC-volume	0.70
CC-mask	0.73
B factors ($Å^2$ mean)	
Protein	51.04
Ligand	64.55
R.m.s deviations	
Bonds length (Å)	0.012
Bonds Angle (˚)	1.067
MolProbitiy score	2.03
Clashscore	9.35
Rotamer outliers (%)	0.00
Ramachandran plot statistics (%)	
Favored (%)	90.54
Allowed (%)	9.46
Outlier (%)	0.00

Supplementary References

1 Seidel, S. A. *et al.* Label-free microscale thermophoresis discriminates sites and affinity of protein-ligand binding. *Angew Chem Int Ed Engl* **51**, 10656-10659, doi:10.1002/anie.201204268 (2012).

2 Magnez, R., Bailly, C. & Thuru, X. Microscale Thermophoresis as a Tool to Study Protein Interactions and Their Implication in Human Diseases. *Int J Mol Sci* **23**, doi:10.3390/ijms23147672 (2022).

3 Punjani, A., Rubinstein, J. L., Fleet, D. J. & Brubaker, M. A. cryoSPARC: algorithms for rapid unsupervised cryo-EM structure determination. *Nat Methods* **14**, 290-296, doi:10.1038/nmeth.4169 (2017).

4 Punjani, A. & Fleet, D. J. 3DFlex: determining structure and motion of flexible proteins from cryo-EM. *Nat Methods* **20**, 860-870, doi:10.1038/s41592-023-01853-8 (2023).

5 van Heel, M. & Schatz, M. Fourier shell correlation threshold criteria. *J Struct Biol* **151**, 250-262, doi:10.1016/j.jsb.2005.05.009 (2005).

6 Beckers, M. & Sachse, C. Permutation testing of Fourier shell correlation for resolution estimation of cryo-EM maps. *J Struct Biol* **212**, 107579, doi:10.1016/j.jsb.2020.107579 (2020).

7 Emsley, P., Lohkamp, B., Scott, W. G. & Cowtan, K. Features and development of Coot. *Acta Crystallogr D Biol Crystallogr* **66**, 486-501, doi:10.1107/s0907444910007493 (2010).

8 Adams, P. D. *et al.* PHENIX: a comprehensive Python-based system for macromolecular structure solution. *Acta Crystallogr D Biol Crystallogr* **66**, 213-221, doi:10.1107/s0907444909052925 (2010).
